# From hopeless thoughts to suicidal attempt: a topographic analysis of adolescents’ suicidal process using structural equation modeling

**DOI:** 10.3389/fpsyg.2026.1725675

**Published:** 2026-02-26

**Authors:** Héctor Morillo-Sarto, Séfora Ene-Gimeno, Javier Torres-Vallejos, Samara Sáez-Martínez, Joel Juarros-Basterretxea

**Affiliations:** 1 Department of Psychology and Sociology, Universidad de Zaragoza, Zaragoza, Spain; 2 Child and Adolescent Psychology, Asociación AFDA, Zaragoza, Spain; 3 School of Psychology, Universidad Santo Tomas, Santiago de Chile, Chile

**Keywords:** adolescents, psychological distress, structural equation modeling, suicidal attempt, suicide-related behaviors

## Abstract

This study examined adolescents’ suicidal process by considering suicidal-related behaviors as related but distinct elements of the suicidal process, which in turn can explain the association between psychological distress and suicidal attempts. Participants were 918 secondary education students between 14 and 21 years old. Suicidal-related behaviors were measured using Paykel’s Suicide Scale, which includes hopeless thoughts about life’s worth, the wish to be dead, thoughts about taking one’s life, suicidal planning, and suicidal attempts. Psychological distress was measured using the depression and anxiety subscales of DASS-21. Structural equation modeling was used to test the direct and indirect effects of psychological distress on suicidal attempts. Two pathways predominantly explain the effect of psychological distress on suicidal attempts: one through the complete process and another, more direct, from the hopeless thoughts and wish to be dead to the attempt. Hopeless thoughts about life’s worth and wishing to be dead have been demonstrated as crucial factors in the explanation of adolescents’ suicidal attempts. Interventions to prevent adolescents’ suicidal attempts should focus not only on explicit suicidal ideation but on less intense life and death-related ideas like hopeless thoughts and the wish to be dead.

## Introduction

Suicide is the fourth leading cause of death among adolescents and young adults between 15 and 29 years old (World Health Organization [WHO], 2021, 2023). Its global prevalence and the severity of its consequences, from psychological and physical injuries to death, have made suicide one of the most relevant social and health concerns worldwide. Indeed, suicide has been included in the WHO Mental Health Action Plan 2013–2030 and the Sustainable Development Goals (World Health Organization, 2023).

The vulnerability of adolescents to carry out suicidal and suicidal behaviors has been linked to the critical nature of this developmental stage (Glenn et al., 2020; Miller and Prinstein, 2019). Adolescence is a critical period characterized by profound changes, instability, and exploration, where the foundations of the future coherent identity are laid. The formation of who one is and who one wants to be (i.e., life goals and sexual orientation, among others), together with heightened emotional reactivity, can result in an aggravated experience of psychological distress and suffering.

When adolescents’ coping mechanisms are overwhelmed by emotional intensity, it can lead to the use of dysfunctional coping strategies (Morillo-Sarto et al., 2025), increasing the likelihood of suicidal behavior (Glenn et al., 2020). Therefore, the changing and distressing nature of this period (Palmeroni et al., 2020), the early onset of suicide-related behaviors (Voss et al., 2019), and suicide-related behaviors during adolescence correlate with future suicidal behavior (Reinherz et al., 2006), making it particularly relevant for present and future risks to individuals’ well-being. In this context, a deeper understanding of the transition from suffering and hopelessness to suicidal attempts is crucial (Dhingra et al., 2022; Klonsky and May, 2014), especially during vulnerable life stages like adolescence (Glenn et al., 2020).

Research suggests that suicide attempts and suicide are preventable in many cases (Darvishi et al., 2023; Kiran et al., 2023), but there is still no clear explanation why some ideators never attempt suicide while others transition to attempts. Concerning this, it is necessary to examine the entire process from its origin, attending to different types of thoughts about life, death, and suicide, and their transition to suicidal attempt or suicide. A behavior-level analysis of the suicidal process permits a better understanding of potential routes to a suicidal attempt that will benefit the prevention and interception efforts (Favril et al., 2023; Haregu et al., 2023; Klonsky, 2020; Malhi et al., 2018; O'Connor and Kirtley, 2018; Tan et al., 2018).

The attention to different specific behaviors related to suicide complements the research relying on latent constructs of suicidal-related behaviors (e.g., summing multiple items into a general factor or suicidal ideation or two factors of passive and active suicidal ideation). This behavior-specific approach seeks to analyze suicide-related behaviors as interrelated discrete behaviors, giving alternatives to researchers in the area. For example, it allows us to research the effect of different elements (e.g., psychological distress) on specific suicidal-related behaviors (e.g., wish to die) instead of a latent construct of suicidality (de Beurs et al., 2021). Similarly, when this approach is applied to the suicidal process, permits making critical distinctions among different suicidal-related behaviors (e.g., suicidal thoughts [wish to die, thoughts of taking one’s life, suicidal planning]) that may help to put into context the pathways to suicidal attempt or even detect the more nuanced ones.

**Figure 1:**
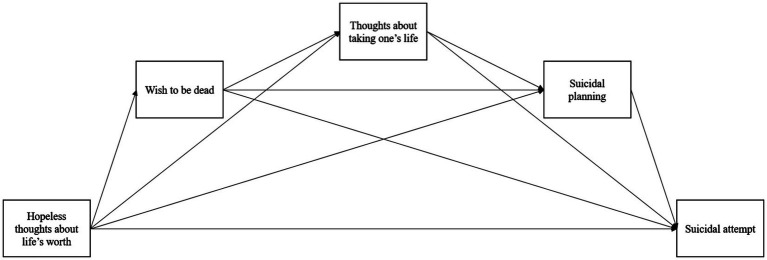
Potential paths of the suicidal process.

Based on behavior-level analysis, Paykel et al. (1974) analyzed in their classic study the prevalence of five suicide-related behaviors. Based on their results, they suggested the existence of a continuum, starting from thoughts of hopelessness about life’s worth (less severe intensity thoughts) to wishing to be dead, thoughts about taking life, and planning suicide (more severe intensity thoughts) that can precede suicidal attempts. According to the authors, this continuum would represent a general tendency where thoughts escalate through a sequence. Still, they also acknowledged that not every individual who follows this sequence makes a suicidal attempt, and even among those who attempt to do so, this sequence may not necessarily be followed precisely, and other alternative pathways can explain suicidal attempts. This way, Paykel et al. (1974) inferred a predominant way from hopelessness to a suicidal attempt while allowing for alternative pathways (Figure 1).

Although Paykel et al. (1974) did not directly analyze the continuum or alternative path or frame their explanation as a theory, their work tentatively captures relevant elements, some of which are directly aligned with recent theories based on the ideation-to-action framework (Joiner, 2005; Klonsky and May, 2015; O'Connor, 2011; O'Connor and Kirtley, 2018; Van Orden et al., 2008, 2010). First, the progressive nature of the suicidal process, where more intense or elaborated suicidal thoughts are often preceded by less intense or elaborated suicide-related thoughts, which ultimately increases the likelihood of a suicidal attempt. Second, the specific-behavior approach allowed Paykel et al. (1974) to note that suicidal attempts are generally preceded by suicidal ideation, but suicidal ideation does not always predict a suicidal attempt. This finding is consistent with the differentiation of the progression to ideation from the ideation-to-action transition. Third, even with the predominance of the general continuum, other potential routes are likely to predict suicidal attempts congruently with non-zero risk behaviors and alternative paths acknowledged in the ideation-to-action framework. Fourth, the relationship of hopeless thoughts with suffering and its role as a catalyst for ulterior suicidal ideation and attempt. Nevertheless, Paykel et al. (1974) have rarely been analyzed in light of current theories and congruent methods (see, for example, Fonseca-Pedrero, 2018; Fonseca-Pedrero et al., 2020).

A behavior-based analysis of suicide-related behaviors, such as Paykel et al.’s (1974) differentiation of discrete behaviors, has been demonstrated to help understand the phenomenon better. This approach has been thoroughly developed using network analysis and has advanced our knowledge of associative patterns between these suicide-related behaviors (De Beurs et al., 2021; Fonseca-Pedrero, 2018; Fonseca-Pedrero et al., 2020). Despite the data-driven exploratory nature and non-directional focus of network analysis being proper, especially in the absence of well-developed theories, it limits its utility to test models based on hypothetical directionality. Alternatively, structural equation modeling (SEM) provides a theory-driven framework to evaluate directional hypotheses. Thus, SEM allows to test the hypothetic model of Paykel et al. (1974), but also tests whether alternative routes exist, consistent with the acknowledgement of non-linearity (e.g., hopelessness effect on suicidal attempt through the wish to die), and even the role of suicidal planning as a mediator between less elaborated ideation and suicidal attempt (e.g., aligned with Integrated Motivational-Volitional Model) by estimating indirect effects.

The current research has three aims. The first aim is to topographically analyze the relationship between life and death-related thoughts (hopeless thoughts about life’s worth, passive and active suicidal ideation) and suicidal attempts among adolescents, considering all possible effects. We hypothesize that suicidal attempts will be predominantly explained by the complete process from hopeless thoughts to the wish to be dead, unspecified suicidal ideation, suicidal planning, and suicidal attempt. Nevertheless, due to the exploratory nature of the study, other paths are not discarded.

The second aim is to analyze the effect of psychological distress on the suicidal process, considering both direct and indirect effects on suicidal attempts. We hypothesize that the effect of psychological distress on suicidal attempts is fully mediated by the transition from hopeless thoughts to more severe suicidal-related behaviors. Specifically, we postulate that the effect of psychological distress on suicidal attempts will be mainly explained by the complete suicidal process. As previously mentioned, considering the exploratory nature of the study, other paths are not discarded.

The third aim is to analyze the effect of group belonging (biological sex [male–female] and collective [heteronormative-LGBTIQ+]) on the model. We hypothesize that the model will be the same for both sexes and collectives.

## Method

### Design

This study employed a quantitative design, utilizing a non-probabilistic convenience sampling. Participants were recruited from educational institutions that agreed to voluntarily collaborate in this research (*k* = 8). Inclusion criteria specified that participants must be a minimum age (14 years) and have cognitive ability (i.e., be able to read and understand the questions and to complete a self-report assessment survey).

### Participants

The study included 918 secondary education students aged 14 to 21 (*M* = 16.51; *SD* = 2.8). 52.7% (*n* = 484) were male, and 47.3% (*n* = 434) were female. 68.8% (*n* = 595) perceived themselves as of medium socioeconomic level, while 20.5% (*n* = 188) and 10.1% (*n* = 93) identified as medium-low and medium-high socioeconomic level, respectively. Only 2.9% (*n* = 27) and 1.6% (*n* = 15) reported low or high socioeconomic status. Detailed descriptive information is displayed in Table 1.

**Table 1:** Descriptive statistics of the sample.

Categorical variables	% (*n*)
Biological sex	
Male	52.7 (484)
Female	47.3 (434)
Collective belonging	
Heteronormative	80.7 (741)
Male	57.9 (429)
Female	42.1 (312)
LGBTIQ+	19.3 (177)
Male	31.1 (51)
Female	68.9 (122)
Suicide-related behaviors	
Felt life not worth living	32.7 (300)
Wished oneself dead	27.1 (249)
Thoughts about taking life	25.7 (236)
Made plans on how to go about taking life	16.1 (148)
Suicidal attempt	12.5 (115)

Continuous variables	*M* (*SD*)
Age	16.5 (2.8)
Psychological distress	10.2 (9.5)

The sample size had a maximum error of 3%, assuming maximum variance and a 95% confidence level. Non-response bias was minimal, with fewer than 1% of students declining to participate. Furthermore, the sample was balanced in terms of biological sex and high school type (public vs. semi-private/charter), ensuring diversity in socioeconomic background and contextual characteristics.

### Procedure

The current study was approved by the Ethics Committee of Aragón (CEICA) (C.I. PI23/379) and the Data Protection Unit of the University of Zaragoza (UPD code: 2023–223).

Data collection dates were agreed upon with each Secondary School representative. Once scheduled, the data were collected during regular tutorial classes for minimal disruption to students’ academic itineraries. All participants were informed about the study’s purpose and confidentiality procedures. They were assured that their participation was voluntary and that they could withdraw without consequences. Due to the sensitive nature of the questions, the importance of maintaining silence and respecting personal privacy during the assessment was emphasized.

Before responding to the questionnaires, all participants accepted the privacy policy and participation in the study. Once informed consent was obtained, participants accessed the online questionnaire and completed the self-report assessments, using their smartphones or computers if available. A SurveyMonkey survey facilitated the data collection by following a direct link or scanning a QR code.

At least one trained researcher was present in each class to supervise the process, ensuring that participants completed the assessment correctly and addressed any questions that might arise during the session.

Following the CEICA’s requirement, data collection was not anonymous, and students could ask for psychological support if required. This support was provided by the Association for the Support of Anxiety and Depression Treatment in Aragón (AFDA), and it was free for participants. To guarantee participants’ privacy, numerical codes replaced the identification data in such a way that any member of the team performing the analysis would not be able to identify the data. The codes will be stored in a separate database with the identification data in case they are needed for intervention.

### Measures

Suicidal related behaviors. The occurrence during the previous year prior to the evaluation of five discrete suicide-related behaviors proposed by Paykel et al. (1974) was evaluated: hopeless thoughts of life’s worth, wish to be dead, thoughts about taking life, suicidal planning and suicidal attempt. The response scale of the items was dichotomic (yes/no).

Psychological distress. Depression and anxiety subscales of DASS-21 (Bados et al., 2005) were used to estimate psychological distress. Due to the consistent evidence of the overlap between depression and anxiety and its essentially unidimensional nature (Luciano et al., 2020; Yeung et al., 2020), the distress score was composed of all depression and anxiety items combined in a unidimensional factor.

#### Biological sex

Collective belonging (LGBTIQ+ or heteronormative). Gender identity (feminine, masculine, other), sexual orientation (heterosexual, homosexual, other), and biological sex (male and female) were used to determine the collective belonging.

### Data analysis

**Figure 2:**
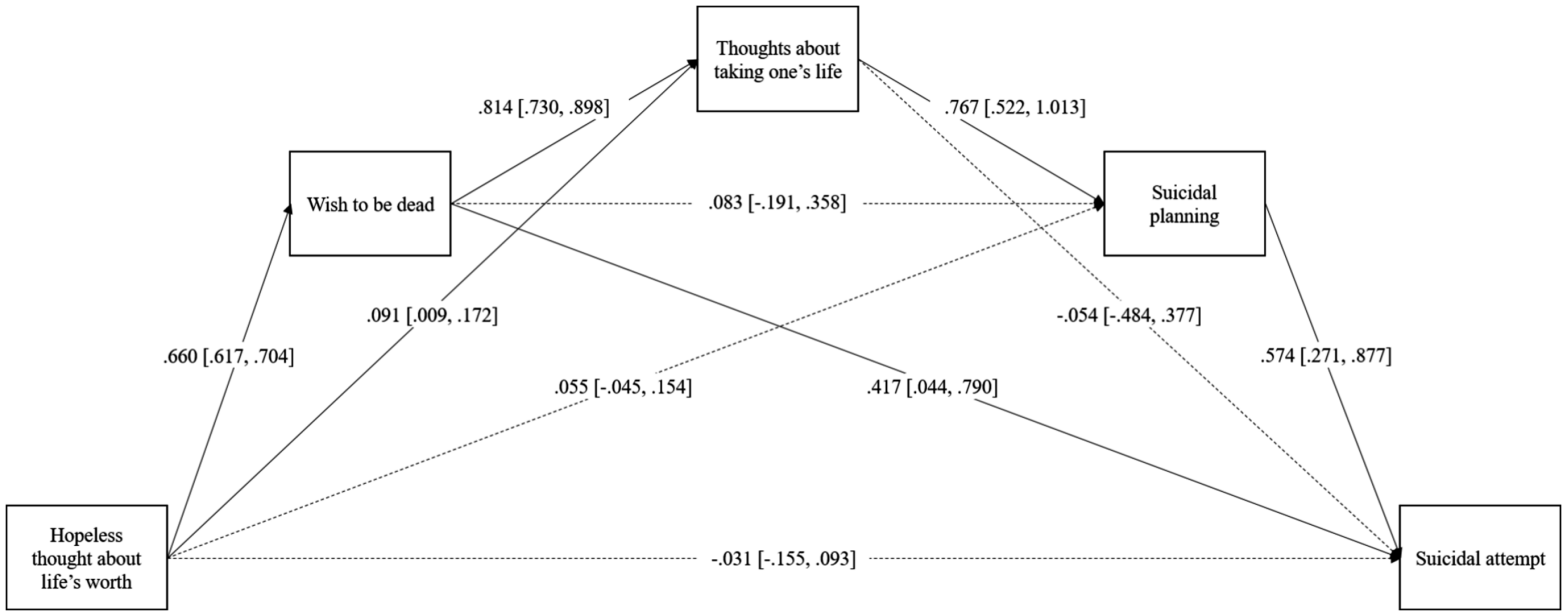
Standardized direct effects of the fully saturated model (Model 1).

Descriptive statistics were calculated for categorical and continuous variables. Frequencies and percentages were used for categorical variables, while means and standard deviations were calculated for continuous variables. Detailed demographic and outcome data are shown in Table 1. To address the non-normality in the categorical data and ensure robust parameter estimates, SEM was conducted using the weighted least squares mean and variance adjusted (WLSMV) estimator (Li, 2016). Bootstrapping with 5.000 resamples was also employed to derive bias-corrected confidence intervals for standardized parameters, improving the accuracy of indirect effects and mediation paths (Preacher and Hayes, 2008; Tibbe and Montoya, 2022). All models were specified with theta parameterization. To reach the study’s first aim, a fully saturated structural equation model was estimated (Model 1). Fully saturated models do not have degrees of freedom, and thus, model goodness of fit tests cannot be conducted. The relevance and interest of these models are the analysis of all the possible relations between all model variables (see Figure 2), allowing the exploratory analysis of all direct and indirect effects. For the second and third aims, the structural equation model was estimated based on the first aim’s results; for the second objective (Model 2), a model to assess the direct and indirect effects of psychological distress on suicidal process elements was calculated. The fitting to the data was evaluated by using the chi-square statistic, the comparative fit index (CFI > 0.95), and root mean square error of approximation (RMSEA < 0.06) and its 95% confidence interval [CI] (Hu and Bentler, 1999). For the third aim, the equivalence of the estimated model for different comparison groups based on the biological sex (male–female) and heteronormativity (heteronormative – LGBTIQ+) was tested by using multigroup analysis, and the model was compared to the nested model where factor loadings and path estimates were constrained to be equal. Mplus 8.7 (Muthén and Muthén, 1998-2017) was used for estimation of the models.

## Results

Descriptive statistics of the sample are displayed in Table 1.

### A fully saturated model of the suicidal process

First, a fully saturated model of suicidal-related behaviors and suicidal attempts was estimated. Due to its exploratory nature, all the possible paths in the model were estimated. The direct effects of the model and their 95% confidence intervals are displayed in Figure 2. Each of the assessed suicide-related behaviors had a statistically significant positive effect (*p* ≤ 05) and thus predicted an increase in the likelihood of the following behavior in the model (e.g., hopeless thoughts predicted a greater wish to die). Of the remaining direct effects, only two were statistically significant: hopeless thoughts increased the likelihood of thoughts about taking life, and the wish to die showed a positive effect on the suicidal attempt.

The indirect effects of Model 1 are summarized in Table 2. Nine indirect effects were statistically significant (*p* < 0.05), five of them on suicidal attempts. As expected, the full pathway involving all the suicidal-related behaviors was statistically significant (path 7). Specifically, hopeless thoughts increased the likelihood of suicidal attempts through sequential increases in the wish to be dead, thoughts about taking one’s life, and suicidal planning. Four additional indirect effects on suicidal attempts followed partial pathways. First, hopeless thoughts about life’s worth increased suicidal attempts via thoughts about taking life and suicidal planning (path 6). Second, hopeless thoughts predicted more suicidal attempts mediated by the wish to be dead (path 1). Third, the wish to be dead predicted the likelihood of suicidal attempts via increased suicidal ideation and planning (path 10). Finally, more suicidal ideation predicted suicidal attempts mediated by an increase in suicidal planning (path 11). The model explained 43.6% of the variance of wishing for death, 76.8% of thoughts about taking one’s life, 76.9% of suicidal planning, and 74% of suicidal attempt.

**Table 2:** Standardized indirect effects of the fully saturated model.

Indirect paths	*β* (95% CI)		
	Thoughts about taking life	Suicidal planning	Suicidal attempt
1. *Hopeless thoughts* through the wish to be dead	**0.470 (0.537, 0.608)**	0.055 (−0.145, 0.222)	**0.275 (0.018, 0.520)**
2. *Hopeless thoughts* through thoughts about taking life	–	**0.069 (0.006, 0.140)**	−0.069 (−0.006, 0.033)
3. *Hopeless thoughts* through suicidal planning	–	–	0.031 (−0.022, 0.113)
4. *Hopeless thoughts* through the wish to be dead and thoughts about taking life	–	**0.412 (0.283, 0.585)**	−0.035 (−0.326, 0.209)
5. *Hopeless thoughts* through the wish to be dead and suicidal planning	–	–	0.031 (−0.093, 0.130)
6. *Hopeless thoughts* through thoughts about taking life and suicidal planning	–	–	**0.040 (0.006, 0.105)**
7. *Hopeless thoughts* through wish to be dead, thoughts about taking life and suicidal planning	–	–	**0.237 (0.104, 0.473)**
8. *Wish to be dead* through thoughts about taking life	–	**0.624 (0.434, 0.879)**	−0.052 (−0.490, 0.317)
9. *Wish to be dead* through suicidal planning	–	–	0.048 (−0.142, 0.199)
10. *Wish to be dead* through thoughts about taking life and suicidal planning	–	–	**0.358 (0.160, 0.709)**
11. *Thoughts about taking life* through suicidal planning	–	–	**0.440 (0.197, 0.836)**

Statistically significant (*p* < 0.05) indirect effects are in bold.

### The effect of psychological distress on suicidal process elements

**Figure 3:**
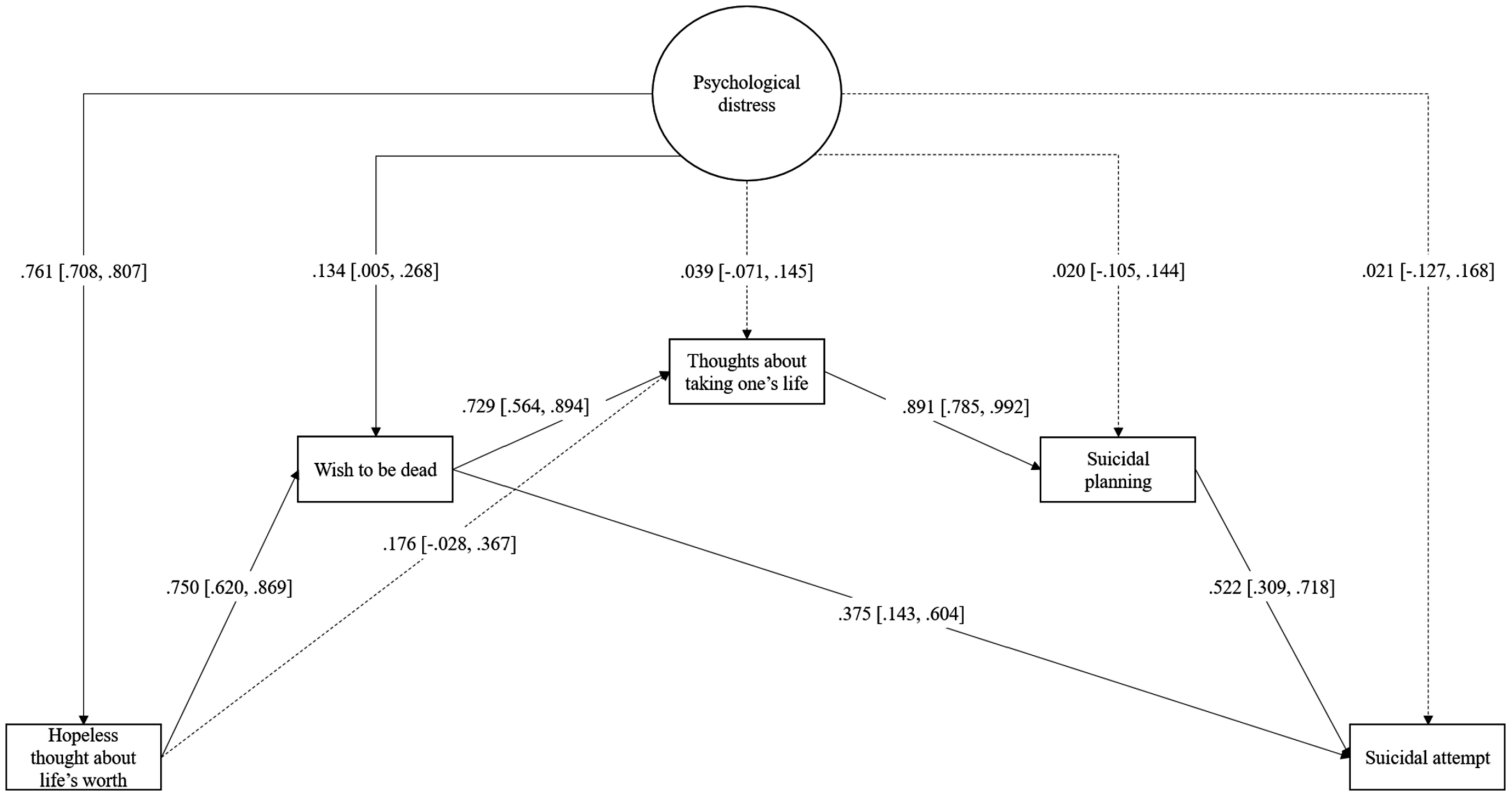
Standardized direct effects of psychological distress on suicidal process elements (Model 2).

To analyze the effect of psychological distress on the different elements of the suicidal process and, ultimately, on suicidal attempts, Model 2 (see Figure 3) was estimated. Based on previous saturated model results, only statistically significant paths were maintained. The model showed appropriate fit to the data (χ^2^_(146)_ = 954.061, *p* < 0.001, CFI = 0.96, RMSEA = 0.08, 90% CI [0.07, 0.08]).

Psychological distress only showed a statistically significant (*p* < 0.05) positive direct effect on hopeless thoughts and the wish to be dead. The remaining effect of psychological distress on suicidal ideation, suicidal planning, and suicidal attempt was indirect, mediated by hopeless thoughts and the wish to be dead (two indirect effects) or only the wish to be dead (two indirect effects).

The indirect effects of Model 2 are summarized in Table 3. Of the 10 statistically significant (*p* < 0.05) indirect effects involving psychological distress, four were on suicidal attempts. As expected, psychological distress exerted an indirect effect on suicidal attempts through the full pathway of suicidal-related behaviors, ranging from hopeless thoughts to suicidal planning (path 6). Three additional indirect effects followed partial pathways; psychological distress influenced attempts via all suicide-related behaviors except hopeless thoughts, beginning with the wish to die (path 9). The final two indirect effects followed shorter pathways: one mediated by hopeless thoughts and the wish to die in one (path 2), and the other solely mediated by the wish to die in the last (path 7).

**Table 3:** Standardized indirect effects of psychological distress on suicidal-related behaviors (Model 2).

Indirect paths	*β* (95% CI)			
	Wish to be dead	Thoughts about taking life	Suicidal planning	Suicidal attempt
*Distress via hopelessness about life’s worth*				
1. *Distress* through the hopeless thoughts	**0.571 (0.465, 0.683)**	0.134 (−0.020, 0.285)	–	–
2. *Distress* through the hopeless thoughts and wish to be dead	–	**0.416 (0.312, 0.563)**	–	**0.214 (0.085, 366)**
3. *Distress* through hopeless thoughts, thoughts about taking life	–	–	0.035 (−0.062, 0.132)	–
4. *Distress* through hopeless thoughts, wish to be dead and thoughts about taking life	–	–	**0.371 (0.271, 0.509)**	–
5. *Distress* through hopeless thoughts and thoughts about taking life and suicidal planning	–	–	–	0.062 (−0.004, 0.147)
6. *Distress* through hopeless thoughts, wish to be dead, thoughts about taking life and suicidal planning	–	–	–	**0.193 (0.110, 0.319)**
*Distress via wishing to be dead*				
7. *Distress* through the wish to be dead	–	**0.098 (0.008, 0.207)**	–	**0.050 (0.006, 0.130)**
8. *Distress* through the wish to be dead and thoughts about taking life	–	–	**0.087 (0.006, 0.185)**	–
9. *Distress* through the wish to be dead, thoughts about taking life and suicidal planning	–	–	–	**0.046 (0.005, 0.113)**
*Distress via thoughts about taking life*				
10. *Distress* through thoughts about taking life	–	–	–	0.018 (−0.029, 0.078)

Statistically significant (*p* < 0.05) indirect effects are in bold.

When compared, some indirect effects were greater than others. Specifically, the complete pathway (path 6) was statistically greater than pathways 9 (Δꞵ = 0.147, 95% CI [0.033, 0.261]) and 7 (Δꞵ = 0.143, 95% CI [0.024, 0.262]), but did not differ from path 2 involving only hopeless and wish to be dead as mediators (Δꞵ = 0.021, 95% CI [−0.152, 0.194]). Similarly, pathway 2 was greater than pathways 9 (Δꞵ = 0.168, 95% CI [0.019, 0.317]) and 7 (Δꞵ = 0.164, 95%CI [0.012, 0.316]). Finally, pathways 7 and 9, absent hopeless thought, did not differ significantly (Δꞵ = 0.004, 95% CI [−0.076, 0.084]).

The R model explained 57.9% of the variance of hopelessness about life’s worth, 73.4% of wishing for death, 83.4% of thoughts about taking one’s life, 81.9% of suicidal planning, and 76% of suicidal attempt.

### The effect of biological sex and belonging to the LGBTIQ+ collective on psychological distress and suicidal process elements

To test the potential effect of belonging to groups defined by the biological sex (male–female) and heteronormativity (heteronormative-LGBTIQ+), multigroup analysis was carried out for each of the categorizations. Results indicated that the constrained model with factor loadings and paths set to be equal fitted well to the data for males and females (*χ* ^2^_(11)_ = 12.806, *p* = 0.31; CFI = 0.97; RMSEA = 0.06, 90%CI [0.05, 0.06]) and heteronormative and LGBTIQ+ groups (χ ^2^_(11)_ = 6.086, *p* = 0.87; CFI = 0.97; RMSEA = 0.06, 90%CI [0.06, 0.07]).

For the male group, the model explained 58.6% of the variance of hopelessness about life’s worth, 74.7% of wishing for death, 83.9% of thoughts about taking one’s life, 82.3% of suicidal planning, and 76.3% of suicidal attempt. Similar results were found for the female group, for whom the model explained 54.2% of hopelessness about life’s worth, 72.5% of wishing for death, 82.5% of thoughts about taking one’s life, 81% of suicidal planning, and 74.7% of suicidal attempt.

For the normative group, the model explained 54.8% of the variance of hopelessness about life’s worth, 71.3% of wishing for death, 81.9% of thoughts about taking one’s life, 80.1% of suicidal planning, and 74.2% of suicidal attempt. Similarly, for the LGBTIQ+ group, the model explained 58.7% of hopelessness about life’s worth, 73.4% of wishing for death, 83.2% of thoughts about taking one’s life, 81.2% of suicidal planning, and 75.6% of suicidal attempt.

## Discussion

Using comprehensive data from 918 secondary school students aged 14–21 years, this study aimed (1) to topographically analyze the relationship between hopeless thoughts about life’s worth, wish to be dead, thoughts about taking life, suicidal planning and suicidal attempts among adolescents, considering all possible effects; (2) to analyze the effect of psychological distress on the suicidal process elements, considering both direct and indirect effects on suicidal attempts; and (3) to analyze the effect of group belonging (biological sex [male–female] and collective [heteronormative-LGBTIQ+]) on the model. Using structural equation modeling to test a theory-driven directional hypothesis, our findings align and validate previous research and help to refine existing knowledge on the suicidal process.

The first hypothesis concerning that suicidal attempts will be predominantly predicted through the complete process was partially supported, while the second hypothesis about full mediation of hopeless thoughts was rejected. Our results confirm the sequential progression from hopeless thoughts about life’s worth to the wish to die, thoughts about taking one’s life, and suicidal planning in the pathway to suicidal attempts as psychological distress increases. Such evidence supports the “suicidal continuum” reflecting the progression from less intense suicidal-related behaviors to suicidal attempts suggested by Paykel et al. (1974) and thoroughly present in contemporary theories (Joiner, 2005; Klonsky and May, 2015; O'Connor, 2011; O'Connor and Kirtley, 2018; Van Orden et al., 2008, 2010). Notably, our results also support these authors’ emphasis on the potential existence of alternative pathways.

In addition to the mentioned complete process, three alternative routes from psychological distress to a suicidal attempt were found. An inspection of routes reveals some similarities and differences; for example, according to the number of suicide-related behaviors present in the paths, two “long” and two “short” pathways are identifiable. Among the long pathways to suicidal attempt, one is the complete process mentioned above, while the second is the same except for hopeless thoughts. In this second route, psychological distress would predict a suicidal attempt through an increase in the wish to die, thoughts about taking one’s life, and suicidal plans. Regarding short pathways, the first one involves hopeless thoughts about life’s worth and the wish to be dead, mediating psychological distress evolution on a suicidal attempt, and the second route only consists of the wish to die. Nevertheless, the difference among routes was not based on the length but on the elements on the pathway: routes involving hopeless thoughts were similar but significantly stronger than pathways not involving it, which were similar among them.

Overall, the results align with the core elements of the ideation-to-action framework as far as the direct predictors of suicidal ideation are not the same of the suicidal attempt. However, a detailed analysis suggests that these findings strongly support certain theoretical propositions over others. Although all ideation-to-action theories acknowledge the potential existence of multiple pathways, their complexity and the specific topography proposed by each differ. The first points of divergence lie in the roles of hopeless thoughts and the wish to be dead. For example, the Interpersonal-Psychological Theory of Suicide (IPTS; Joiner, 2005; Van Orden et al., 2008, 2010) generally posits that the simultaneous experience of thwarted belongingness and perceived burdensomeness as sources of distress (Bhardav and Swords, 2022) and the hopeless expectation of change would strive on active suicidal ideation (e.g., thoughts about taking one’s life and suicidal planning), which would turn in a suicidal attempt in the context of acquired capability. In other conditions (e.g., only perceived burdensomeness), only passive suicidal ideation (e.g., wishing to be dead) would appear, considering it a lower-risk context for suicidal attempt (Van Orden et al., 2010). Alternatively, the Three-Step Theory (3ST; Klonsky and May, 2015; Klonsky et al., 2018, 2021) maintains that pain (usually psychological) and hopelessness are required to cause suicidal desire (Step 1), first modest/passive that could escalate to strong/active as the pain overwhelms connectedness (Step 2), progressing to suicidal attempts if capability is present (Step 3). In line with these proposals, our results confirm the importance of hopeless thoughts, as it defines the most robust pathways, but challenge the secondary role ascribed to the wish to be dead in the IPTS and the requirement of simultaneous presence of pain and hopeless thoughts established in the 3ST.

In contrast to IPTS, the wish to be dead appears central in our population, being a key mediator in all the pathways, particularly in shorter routes to suicidal attempt. Contrary to IPTS predictions, previous research has demonstrated that acquired capability is not specific to suicidal attempts and predicted suicidal ideation (for a review, see Ma et al., 2016). More specifically, recent research has demonstrated that acquired capability prospectively predicted the wish to be dead, which in turn predicted thoughts about taking one’s life (Kivelä et al., 2024). Despite Kivelä et al. (2024) not assessing suicidal attempts, their results may explain ours and how some individuals lead to suicidal attempts transitioning to active suicidal ideation or even only wishing to be dead, in a different route from hopelessness. According to the authors, this progression may occur due to participants’ prior suicide attempts, arguing that individuals without such a history are unlikely to progress from passive to active ideation (Kivelä et al., 2024). However, recent work highlights IPTS’s limitations in predicting attempts among adolescents (Pagliaccio et al., 2024), and these results may support the potential misdefinition of the theory (Chao-Cheng and Linscott, 2022; Espinosa-Salido et al., 2020; Ma et al., 2016; Stewart et al., 2015).

Less clear is how these results align with the 3ST; although this theory does not explicitly address transitions from the wish to die to attempts, the authors did not directly relegate the wish to be dead to a secondary role. Regardless, the results supporting the acquired capability effect on the wish to be dead are generalizable to 3ST postulates, and the obtained results support the plausibility of rapid pathways in our model, even among adolescents without prior suicidal behavior.

Diverging from 3ST, our results suggest that psychological suffering and hopeless thoughts interact but are not both required to cause the wish to be dead. The combined effect is more substantial in explaining suicidal attempts, reinforcing the relevance of the pain and hopeless thoughts in the suicidal process. Nevertheless, alternative routes better explained by other factors challenge the strict requirement of the combined presence of pain and hopelessness. These results are in line with previous research on adolescents showing that hopelessness as well as psychological pain predicted suicidal ideation beyond the interaction among them (Dhingra et al., 2019; Schimanski et al., 2025; Yang et al., 2019), suggesting that the excessively restrictive approximation is not coherent with the complex nature of the suicidal process.

Contrary to the ITPS and the 3ST, these findings strongly support the Integrated Motivational-Volitional Model (IMV; O'Connor, 2011; O'Connor and Kirtley, 2018). The IMV is well-suited to explain these results due to its integrative nature. Our findings particularly support the IMV in two ways. First, IMV indicates that future expectations emerge as influential in the relationship between suicidal distress and suicidal ideation/intent, though not exclusively. This finding aligns with the partial mediation of hopeless thoughts in the distress-ideation relationship, as other unmeasured factors (e.g., social support, resilience) may also contribute. Second, the IMV assumes that the transition from ideation/intent to attempts is influenced by multiple factors, such as planning, without limiting it to this element. In our model, planning is critical in two of the four pathways, and the fact that thoughts about taking one’s life only predict attempts through planning further supports the IMV. Within this framework, the direct pathway from the wish to die could be explained by factors like impulsivity, particularly plausible given the adolescent sample (Miller and Prinstein, 2019; Stewart et al., 2015).

Finally, we hypothesize that the model will be the same for both sexes and collectives. The model’s invariance across sexes (male–female) and collectives (heteronormative-LGBTIQ+) implies that the model is the same for the different groups, making them comparable. It is important to note that this result does not reject differences between groups found in previous research (e.g., Hernández-Flórez et al., 2023; Kann et al., 2011; Rosario et al., 2005) as far as they are referred to levels of suicidal risk of psychological distress, but are congruent with those results indicating no moderation and mediation effects (e.g., He et al., 2023; Li et al., 2024).

### Strengths and limitations

The current research includes a large and diverse sample of secondary education students, covering a wide age range and ensuring gender balance and attending to minorities, responding to researchers’ demands in the area (Arango et al., 2021; Liu et al., 2024; Miller and Prinstein, 2019). Additionally, the sample represents different socioeconomic backgrounds, with a majority identifying as medium socioeconomic level and a balanced distribution across school types (public vs. semi-private/charter schools). These factors enhance the study’s external validity and generalizability to adolescent populations in similar educational settings. Furthermore, the non-response bias was minimal, as fewer than 1% of students declined participation, strengthening the reliability of the findings.

While this study provides valuable insights into the behavior-based analysis of the suicidal process in adolescents, certain limitations must be acknowledged. First, cross-sectional designs are useful for identifying associations between psychological distress and suicide attempts as well as between different suicide-related behaviors. This approach allowed for the estimation of an exploratory model of the suicidal process and offered a non-linear, tentative interpretation of the suicidal process. However, the interpretation of findings remains constrained by the cross-sectional nature of the data. Temporal relationships between different suicide-related behaviors and autocorrelations cannot be fully established, and potential fluctuations in psychological distress over time are not captured. Future research should test and discuss this model using longitudinal data (e.g., cross-lagged models, ecological momentary assessment) to better assess the dynamic nature of suicidal processes. Second, we used self-reported measures, considering that it is the only way to assess large samples. Furthermore, this method has yielded higher reporting of these sensitive nature behaviors than interview-based measures (Liu et al., 2024). Nevertheless, clinical interviews focused on specific samples may provide more accurate data in the future, as the nature or forms of attempts. Addressing these limitations will strengthen the theoretical and empirical understanding of suicidal processes in adolescents.

## Conclusion and implications for research, treatment, and prevention

The results obtained in the current research emphasize the need to assess the transition between suicidal-related behaviors (i.e., from thinking that life has no sense to desiring death and from planning to attempt) as related but distinct processes, instead of collapsing them into a unique variable of suicidal risk (Allan et al., 2023; Paykel et al., 1974). This approximation can explain the modest effects of psychological distress on suicidal ideation and suicidal behavior found in previous research (Franklin et al., 2017) and underline the relevance of different elements of the suicidal process, especially the hopeless thoughts and the wish to be dead. In line with this, results align with recommendations of distinguishing passive ideation from active intent and avoiding a hierarchical approach of using “lower risk” behaviors as screening (Wastler et al., 2023) due to the potential of passive ideation (e.g., “wish to die”) to predict attempts, particularly in adolescents, where impulsivity can override intent.

The results obtained here have important implications for intervention and treatment. In line with previous research (e.g., Kleiman and Beaver, 2013; Miranda et al., 2014) these results direct us to suggest interventions with adolescents that take into account two different aspects of suicidal ideation to prevent adolescents’ suicidal attempts; first, focusing on the elaboration level of the ideation beyond the ideation itself, considering its role as a coping mechanism to affront psychological suffering. Second, the emphasis and necessity to take into account the more direct paths from less elaborated suicidal ideation to suicidal attempt does not minimize the absence of suicidal planning and, thus, the risk of suicidal attempt (Wastler et al., 2023). In both cases, interventions oriented to empower adolescents on problem-solving strategies, emotion regulation, detecting help resources, and help-seeking behaviors could be crucial in the future prevention of suicide attempts (Hetrick et al., 2014).

Finally, these results also have direct implications for preventive programs, especially in school-based approaches and risk-screening tools. Based on these results, we suggest that screening instruments and school protocols should prioritize a nuanced assessment of hopeless thoughts about life and desiring death. Rather than simply asking about suicidal thoughts (e.g., “Have you had suicidal thoughts?”), screening actions in the school context and screening tools should be refined to specifically inquire into the content and elaboration of those thoughts. This granularity can help school team members to identify distinct risk profiles and tailor immediate interventions more effectively.

In conclusion, this study sheds light on distinct pathways that lead from psychological distress to suicidal attempts. By identifying routes that progress through suicidal ideation and planning and more direct paths to attempts, we underscore the importance of developing multifaceted, personalized intervention strategies. Addressing the emotional and cognitive underpinnings of these pathways is crucial for effective suicide prevention, particularly in adolescent populations. Continued research into these processes will be vital in refining interventions and ultimately reducing the incidence of suicidal behaviors in youth.

## Data Availability

The datasets presented in this article are not readily available because the dataset is not publicly available to ensure participant confidentiality. As guaranteed to the participants during the informed consent process, the data will not be shared in whole or in part with anyone outside the core research team. Requests to access the datasets should be directed to joeljuarros@unizar.es.
